# Editorial: Advancing sports cardiology: new frontiers in athlete screening and recovery

**DOI:** 10.3389/fcvm.2026.1883248

**Published:** 2026-06-08

**Authors:** Stefano Palermi, Francesca Graziano, Sara Monosilio, Luna Cavigli, Marco Vecchiato

**Affiliations:** 1 Department of Medicine and Surgery, Saint Camillus International University of Health Sciences, Rome, Italy; 2 Department of Cardiac, Thoracic and Vascular Sciences and Public Health, University of Padova, Padova, Italy; 3 Department of Clinical, Internal, Anesthesiological and Cardiovascular Sciences, Sapienza University of Rome, Rome, Italy; 4 Department of Medical Biotechnologies, University of Siena, Siena, Italy; 5 Department of Medicine, University of Padova, Padova, Italy; 6 Theoretical and Applied Sciences, eCampus University, Novedrate, Italy

**Keywords:** artificial intelligence, cardiovascular screening, digital health, pre participation screening, sports cardiology

Sports cardiology is a rapidly evolving field at the intersection of maximizing athletic performance and safeguarding cardiovascular health (1). With the continuous advancement of diagnostic technologies and the growing diversity of athletic populations, understanding the cardiac impacts of intense physical activity has become increasingly complex (2, 3). This Research Topic was conceived to explore the latest challenges and innovations, emphasizing a balanced approach that integrates technological advancements with athletes' diverse physiological needs (4–7). The five original articles collected herein contribute to shaping new cardiovascular screening methods, exploring the central role of artificial intelligence (AI), defining updated recovery guidelines, and assessing the impact of explosive training.

## Pre-participation screening and the role of artificial intelligence

Preventive cardiovascular evaluation is a fundamental tool for mitigating the risk of sudden cardiac death in athletes, which can be triggered by hidden genetic conditions or coronary abnormalities (8, 9). However, differentiating normal physiological adaptations resulting from intensive training from early pathological alterations remains a major clinical challenge (10, 11). In this context, Banerjee analyzes the evolution of screening protocols, highlighting how integrating digital tools and machine learning—including AI-enhanced stethoscopes and predictive software for ECG and cardiac magnetic resonance—can revolutionize sports cardiology by optimizing diagnostic accuracy and preventing adverse events.

Addressing the intrinsic complexity related to ethnic differences and varying degrees of remodeling in athletic populations (12), Guo and Wu propose an innovative multimodal AI-driven framework. Their system utilizes *CardioSpectra* and *Risk-Stratified Exertional Embedding* (RSEE) models to simultaneously analyze multimodal clinical inputs, such as genomic data, echocardiographic images, and wearable sensor signals. This approach explicitly models exertion-induced variations, drastically reducing false positives and ensuring a clinically interpretable and highly personalized risk stratification (13).

## Return-to-play and long-term adaptations

Another essential frontier is ensuring athlete safety during the post-infectious phase and developing evidence-based return-to-play protocols (14–16). The prospective longitudinal study conducted by Schellenberg et al. fills an important gap by providing reassuring long-term data on athletes who contracted mild forms of SARS-CoV-2 infection. Through a careful 12-month follow-up using advanced echocardiography and cardiopulmonary exercise testing, the research demonstrated that cardiac structure, systolic and diastolic function, and aerobic performance (VO2peak) remain stable. These findings exclude long-term adverse myocardial remodeling and confirm the safety of returning to competition for asymptomatic athletes, showing that their cardiopulmonary tolerance is preserved (17–19).

## Recovery monitoring and performance optimization

Non-invasive monitoring of workload and systemic inflammation is a decisive advantage for preventing overtraining (4, 20). Ramiro-Cortijo et al. examined the relationship between heart rate variability (HRV) and plasma cytokines released in response to physical exertion. The results reveal that greater parasympathetic activation (detected via the HF/TP ratio) at rest and immediately post-exercise is associated with lower levels of exercise-induced pro-inflammatory cytokines. This positions HRV not only as a classic autonomic index but as a promising non-invasive predictor of training-induced inflammation, facilitating the optimization of recovery strategies (21, 22).

In the field of neuromuscular optimization, the systematic review and meta-analysis by Ma et al. examines the specific physical responses to explosive stimuli. The authors demonstrate that plyometric training induces neuromuscular adaptations that significantly improve performance in the counter-movement jump (CMJ) compared to routine training. By fully exploiting the muscle stretch-shortening cycle to generate maximal force, this approach confirms its role as a cornerstone of explosive power development in contemporary athletic preparation (23, 24).

## Conclusion

The articles comprising this Research Topic offer a clear perspective: the “one-size-fits-all” approach to cardiovascular assessment and training prescription is giving way to highly tailored strategies (25). By integrating artificial intelligence, multimodal screening, advanced myocardial deformation metrics, and non-invasive biomarkers for inflammation, physicians and coaches can now distinguish pathology from benign adaptation with far greater precision (6, 26). We thank all the authors and reviewers for their invaluable contributions, which help refine care and forge a sporting environment where elite performance and clinical safety coexist in harmony.
